# Directed Forgetting of Negative Self-Referential Information Is Difficult: An fMRI Study

**DOI:** 10.1371/journal.pone.0075190

**Published:** 2013-10-04

**Authors:** Wenjing Yang, Peiduo Liu, Qian Cui, Dongtao Wei, Wenfu Li, Jiang Qiu, Qinglin Zhang

**Affiliations:** 1 Faculty of Psychology, Southwest University, Chongqing, China; 2 School of Political Science and Public Administration, University of Electronic Science and Technology of China, Chengdu, China; University of California, San Francisco, United States of America

## Abstract

A large body of evidence suggested that both emotion and self-referential processing can enhance memory. However, it remains unclear how these two factors influence directed forgetting. This study speculates that directed forgetting of negative self-referential memory is more difficult than forgetting of other-referential memory. To verify this speculation, we combined the directed forgetting paradigm with the self-reference task. The behavioral result suggested that although both self-referential and other-referential information can be directly forgotten, less self-referential information can be forgotten than other-referential information. At the neural level, the forget instruction strongly activated the frontal cortex, suggesting that directed forgetting is not memory decay but an active process. In addition, compared with the negative other-referential information, forgetting of the negative self-referential information were associated with a more widespread activation, including the orbital frontal gyrus (BA47), the inferior frontal gyrus (BA45, BA44), and the middle frontal gyrus. Our results suggest that forgetting of the self-referential information seems to be a more demanding and difficult process.

## Introduction

In the course of daily life, it is important to set aside outdated or irrelevant information out from the mind and turn to focusing on current tasks. These demands of memory control are often investigated with the paradigms of directed forgetting [1]–[5] and think/no-think (TNT) [6]–[8]. The directed forgetting paradigm is frequently used in cognitive psychology and neuropsychology to determine the ability to voluntarily suppress irrelevant information. This paradigm has two common variants: item and list methods. In the item method, each item is directly followed by a memory instruction, either a “remember” (“R”) instruction or a “forget” (“F”) instruction, and participants are asked to follow the instructions. In the list method, participants are asked to remember a list of presented words for later testing. However, a surprise instruction to forget the preceding words is given halfway through the list. Later, the memory is tested for all the items, regardless of their initial instruction. The directed forgetting effect is obtained when the number of items instructed to be remembered is higher than the number of items instructed to be forgotten during the test period.

When the item method directed forgetting paradigm (the paradigm of interest in the present investigation) was used, the selective rehearsal hypothesis and the attentional inhibition hypothesis were proposed to explain the directed forgetting effect. Previous empirical data supported the selective rehearsal hypothesis, indicating directed forgetting result from differential encoding and rehearsal of to-be-remembered (TBR) and to-be-forgotten (TBF) items [5], [9], [10]. In recent years, a lot of behavioral and neuropsychological evidence favored the attentional inhibition hypothesis [5], [11]–[15]. The attentional inhibition hypothesis states that directed forgetting results from the attentional inhibition of information during encoding. Specifically, the “F” instruction triggers attentional inhibition that terminates the rehearsal of TBF items or suppresses their memory activation to below baseline levels (i.e., representational inhibition) [5], [13]. Mechanisms engaged in attentional inhibition are possibly associated with cognitive control processes akin to those used to control overt actions [12], [13]. Neuropsychological data also documented the inhibition hypothesis of directed forgetting [1], [7], [13], [14], [16], [17]. Event-related potential (ERP) studies suggest that the frontal or prefrontal N2 component elicit by the “F” instruction, which reflects the inhibition process, plays an important role in directed forgetting [16]. In recent years, functional magnetic resonance imaging (fMRI) studies have suggested that frontal cognitive control processes play important role in memory control [1], [7], [8], [13], [17], [18]. Specifically, Wylie et al. (2007) investigated whether intentional forgetting can be viewed as an active process. The fMRI data suggest that the answer is positive and that the frontal control process might have an important role in directed forgetting [1]. Bastin et al. (2012) examined the neural substrates associated with remembering and forgetting at both the encoding and retrieval stages. Encoding TBF items was associated with higher activation in the right middle frontal and posterior parietal cortex, known to intervene in attentional control [18].

An interesting issue in memory control is whether people can intentionally forget self-related emotional memories, particularly negative memories. Considerable evidence have suggested that human brain is especially sensitive to emotionally negative events, and that these events are preferentially processed relative to neutral and positive events from early visual processing and attention allocation to later higher cognitive processing [19]–[23]. So, the ability to control negative events is expected to be limited. Some studies also confirmed this hypothesis and found that people cannot intentionally forget emotionally negative events [24], [25]. However, several empirical studies on directed forgetting of emotional memory indicated that people can still forget emotionally negative memories [4], [26], [27]. Tolin et al. (2002) observed that directed forgetting of pleasant and unpleasant words reveals intentional forgetting of both types [28]. Similarly, several studies on clinical populations also found directed forgetting with trauma-related words [26] or depression-related words [27]. In recent years, studies attempted to investigate the neural mechanism of directed forgetting of negative information [3], [16], [17]. Nowicka et al. (2011) explored the different neural substrates of directed forgetting of emotionally negative events fMRI data suggested that forgetting negative information were associated with widespread activations extending from the anterior to posterior regions. By contrast, forgetting neutral information is associated with only a cluster of activation in the right lingual gyrus [17].

Some behavioral and neural studies investigated the directed forgetting effect of emotional memory. However, to the best of our knowledge, only a few studies investigated whether intentional forgetting can still occur if negative information is enhanced by the self-reference effect (SRE) [27]. It is well documented that self-referential processing yields superior memory compared with information encoded using semantic and other-referential encoding strategies [29], that is the SRE in the memory [30]–[34]. Several researchers argued that SRE appears primarily because the self is a well-developed and often-used construct that promotes elaboration and organization of encoded information [30]. Recent neuroimaging studies have found stronger medial prefrontal cortex (MPFC) and rostral anterior cingulate (ACC) activation linked to self judgments relative to other judgments [31], [35]–[37]. The activation of the MPFC may reflect the self-reflective process and the activation of the ACC may suggest that the participants think of their own physical appearance and generate relevant emotional responses [31], [33]. Self-referential processing can enhance the memory; however, whether people can intentionally control this enhanced memory remains unknown. To the best of our knowledge, only some behavioral studies tried to explore this problem with normal participants [27]. Power (2000) asked participants to process positive and negative information in relation to themselves. The results showed that although healthy students recalled more positive than negative information, both healthy students and “depressed” students showed directed forgetting [38]. Thus, people can still intentionally forget the memory which was enhanced by the self-referential processing. Some other studies investigated the directed forgetting of recently autobiographical memories [39], [40]. The results suggested that both the negative and the positive autobiographical memories can be directed forgotten. However, the neural mechanisms of directed forgetting of self-referential memory remain unknown. Meanwhile, there was no other-referential condition in the previous studies. Thus, the difference between intentional forgetting of self-referential memory and other-referential memory cannot be determined.

This study firstly combined the self-referential task and the directed forgetting paradigm to investigate the difference between directed forgetting of self-referential memory and other-referential memory. In our study, the other public referential person is Luxun, a famous literator in China. Negative memories are the ones usually desired to be forgotten, so we used negative information as stimuli. The following hypotheses were tested. First, previous studies indicated that the ACC and MPFC activated when people think of their own physical appearance and it also linked to oneself emotional self-control during the SRE task,so we expected to find more activation in the MPFC and ACC in self- than in other-judgment conditions [31], [34], [35]. Second, previous studies suggested that intentional forgetting was an active process in which the frontal control was critical [1]; thus, the activations of the frontal areas were expected to be associated with the “F” instruction. Third, because numerous studies suggested that the more difficult the task, the stronger/larger the activations [17], [41], and the self-reference information is enhanced by the self-relative process, so we hypothesized that forgetting of the self-referential information would activate a more widespread neural activations.

## Methods

### Ethics Statement

The experiment was approved by the Academic Committee of the School of Psychology, Southwest University in China. All participants signed an informed consent form prior to their inclusion in the study.

### Participants

Twenty five undergraduate students (14 females, 11 males; aged 19 to 24 years; mean age, 22.5 years) of the Southwest University in China participated in the experiment as paid volunteers. All the subjects were Chinese native speakers with normal or corrected-to-normal vision, and reported no current or past neurological or psychiatric disease.

### Design and Materials

A 2 (instruction: R vs. F) × 2 [reference: self-reference (SR) vs. other-reference (OR)] within-subjects design was used. The stimuli for the experiment were 240 negative trait adjectives consisting of two to four Chinese characters selected from established personality trait adjective pools [42]. The adjectives were classified into two lists of 120 words matched in word length, word frequency, and valence. One list was presented during the study phase; the other was used as foil items during recognition test period. The study and the test lists were counterbalanced. Each word used in the study phase was randomly assigned to one of two subsets: “TBR” and “TBF”. Allocation of items to the TBR and TBF categories was counterbalanced and administered to the participants randomly. The study words were classified into three lists of 40 words, which were presented in one of three blocks.

### Procedures

**Figure 1:**
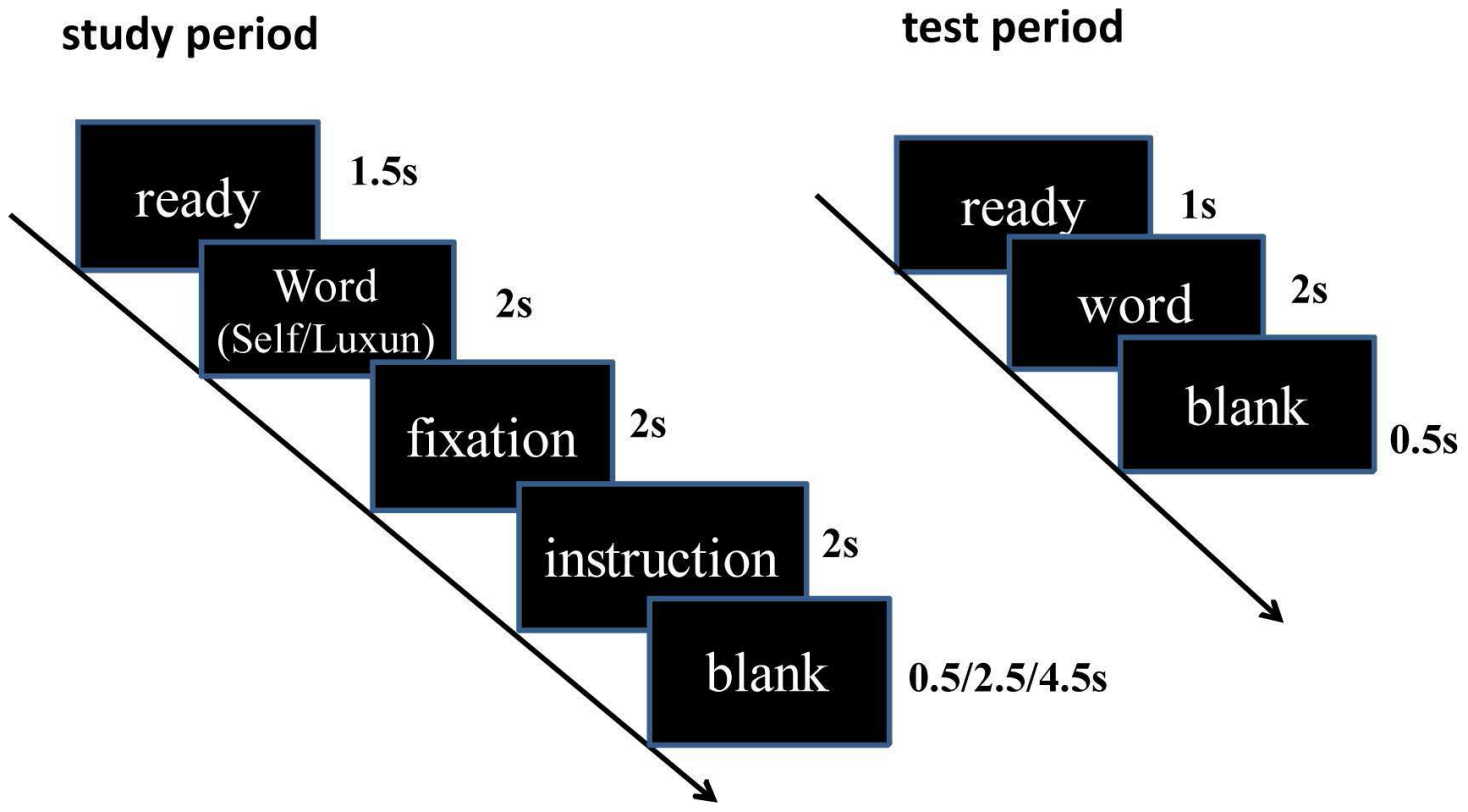
Time sequence of a trial. The left side is a trial of the study phase and the right shows a trial of the test phase.

The experiment was divided into two phases: study and test (see Figure 1). The subjects were scanned only at the study phase. Prior to entering the scanner, all participants were instructed to practice the study procedure until they know their task. The study phase comprised three runs, each of which consists of 40 trials. Each trial was initiated by the prompt “ready” lasted for 1.5 s. One of the 120 study words was then presented on the screen, during which the participants were required to judge if an adjective was proper to describe the reference people (self or Luxun, a well-known Chinese literator). The adjective word was only presented for 2 s and participants need to make a decision during this period. If the participants did not respond during this period, the word will disappear. After judging, a fixation “+” was presented on the screen for 2 s. The previously assigned instruction (remember or forget) was then displayed for 2 s. The participants were asked to remember only the words followed by the remember instruction while attempting to forget any word followed by the forget instruction. Each trail ended with a random blank that ranged from 0.5 s to 2.5 s to 4.5 s. The order of experimental trials was pseudo-random with the constraint of no more than three consecutive trials with the same type of instruction or the same type of stimulus in sequence. In our study, no jitter existed before the memory instruction. Therefore, activity associated with the instruction screen would probably include activity that “leaked” from previous events (i.e. word and fixation point screen). However, the reference type and the memory instrucion were presented randomly in our study. If the activity associated with memory instruction would probably include activity that “leaked” from previous events, both the forget instructions and the remember instructions contained this activity. Under this condition, if the brain activity of these two memory instructions were still different, these differences should be attributed to the different memory instructions but not the previous stimuli.

Immediately after the end of the study phase, the recognition test was conducted outside the scanner. All the presented words (TBR or TBF study words) were presented intermingled with an equal number of foils. Each trial of this phase began with the presentation of a prompt “ready” that lasted for 1 s. The words were then presented for 2 s, and the participants were instructed to press one button if the word had been presented during the study phase (old), irrespective of the “F” or “R” instruction, and another button if the word had not been presented previously (new). Before the test, the participants were stressed with the importance of disregarding the previous “R” or “F” memory instruction when the response was made.

### fMRI Data Acquisition

Images were acquired with a Siemens 3T scanner (Siemens Magnetom Trio TIM, Erlangen, Germany) equipped with an eight-channel phased array coil. Head movement was minimized by restraining the participant’s head using a vacuum cushion. Stimuli were displayed on a screen positioned at the rear of the scanner, which the participant can comfortably see through a mirror mounted on the standard head coil. In all the sessions, the first five volumes were discarded to account for the time needed for the field to achieve a steady state.

Functional data were acquired using a T2-weighted gradient echo planar imaging (EPI) sequence [32 slices, voxel size = 3.4 mm × 3.4 mm × 3 mm voxels; TR = 2000 ms; TE = 30 ms; field of view (FOV) = 220×220 mm^2^; flip angle = 90°; matrix size = 64×64]. T1-weighted high resolution anatomical images were also acquired for each participant (176 sagittal slices, TR = 1,900 ms; TE = 2.52 ms; FOV = 256×256; voxel size = 1 mm×1 mm×1 mm).

### fMRI Data Analysis

The fMRI data were preprocessed and analyzed using SPM8 (http://www.fil.ion.ucl.ac.uk/spm/; Wellcome Department of Imaging Neuroscience, London, United Kingdom). Functional images were corrected for slice acquisition time within each volume and motion corrected with realignment to the first volume. These images were then normalized to the MNI EPI template (voxel size, 3×3×3 mm^3^). The normalized data were spatially smoothed with a Gaussian kernel; the full width at half maximum (FWHM) was specified as 8×8×8 mm^3^. A high-pass filter was implemented with a cut off period of 128 s to remove low-frequency drifts from the time series.

After pre-processing, statistical analysis for each individual participant was conducted using the general linear model (GLM) [43]. The BOLD signal was modeled by convolving the design matrix with a canonical hemodynamic response function. A 2 (reference: SR, OR)×2 (memory instruction: R, F) factorial design yield four conditions, namely, self remember (SR), self forget (SF), other remember (OR), and other forget (OF). We modeled the onsets of each of these four conditions and each trial was modeled as a separate event (duration = 0). In addition, six realignment parameters for each subject were also included in the model as confounding factors. For each subject, all the three runs were modeled in a GLM.

All the 25 participants were included in the second-level analysis. The first level analysis of each subject yielded four contrasts (SR, SF, OR, OF) images associated with each condition modeled. Results from the first level analysis were analyzed with an ANOVA. In this ANOVA, we tested for the main effect of the reference and the memory instruction, the interaction of reference type×memory instruction, and the simple effect of the directed forgetting effect of the self-reference information and the other-reference information. For all the analyses, the threshold was set to p<0.001 (FDR corrected)for multiple comparisons with a threshold for minimum spatial extent of 10 contiguous voxels.

## Results

### Behavioral Data

The mean proportion of old recognition rates for old responses made on the recognition test is shown in Table 1. The TBR and TBF stimuli were submitted to an analysis of variance with the reference type (SR and OR) and instruction type (TBR and TBF) as factors. The results revealed a main effect of the instruction type [F (1, 24) = 21.556, P = 0.000, partial η^2^ = 0.407], with TBR items (0.727±0.034) were more often recognized than TBF items (0.618±0.039).The results also showed a main effect of the reference type [F (1, 24) = 25.247, P = 0.000, partial η^2^ = 0.473]. Participants made more old responses to the self-referential items (0.718±0.035) than the other-referential items (0.627±0.036). There was also a significant interaction between the instruction and the reference [F (1, 24) = 4.457, P = 0.048, partial η^2^ = 0.117]. The simple effect analysis showed that directed forgetting of self-referential information [F (1, 24) = 5.23, P = 0.029, partial η^2^ = 0.140] is smaller than that of other-referential information [F (1, 24) = 33.821, P = 0.000, partial η^2^ = 0.514].

**Table 1:** Means and standard errors (Mean±SE) of response rates for different types words.

	Self-reference	Luxun-reference
**forget**	0.67±0.21	0.55±0.19
**remember**	0.76±0.16	0.67±0.19

### fMRI Data

**Figure 2:**
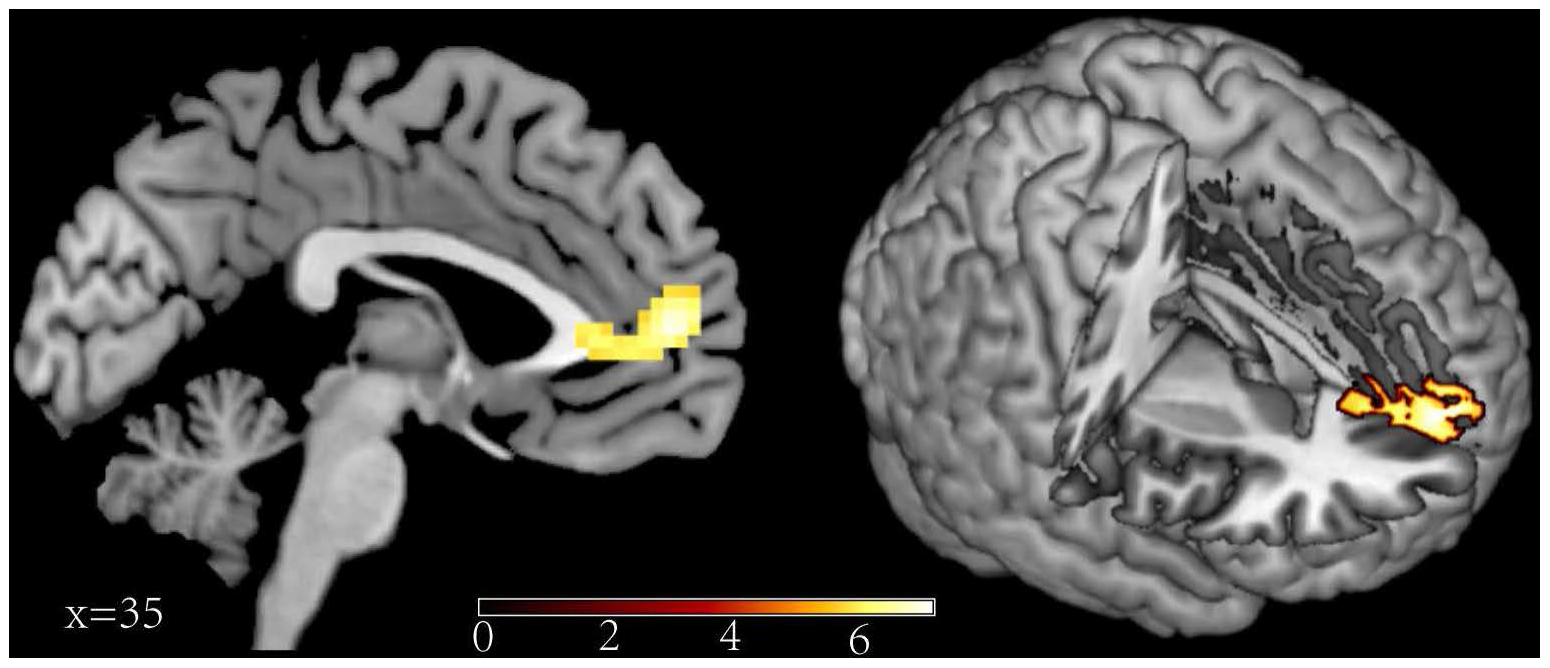
Increased activity of the MPFC and ACC associated with the self-referential relative to other-referential.

**Figure 3:**
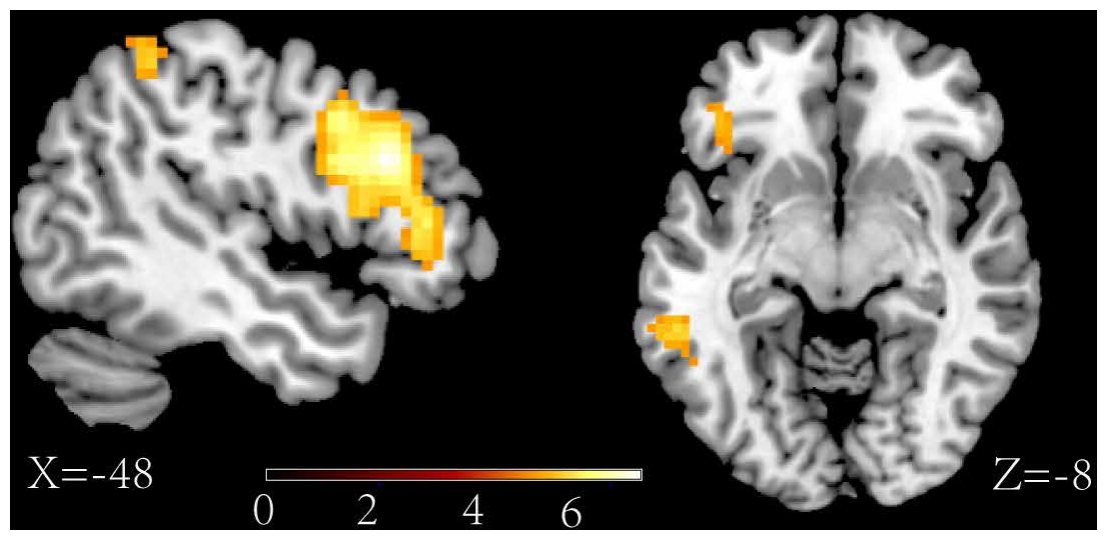
Activations of the forget instruction>remember instruction.

The main aim of this study was to investigate the different neural substrates between directed forgetting of the self-referential memory and the other-referential memory. We must first ensure that experimental manipulation of the reference and the memory instruction was effective, that is the main effect of the ANOVA analysis. Reliable main effect of the reference type and the memory instruction were found. Greater activity for self-referential than other-referential trials was found in the MPFC (BA 10) and ACC (BA 10, BA 25) (see Figure 2, contrast 1 in Table 2). The main result of the instruction showed greater activity for “F” than “R” trials in the middle frontal gyrus (BA 48), inferior frontal gyrus (BA 47), superior parietal lobule (BA 7), inferior parietal lobule (BA 40) and middle temporal gyrus (BA 20, BA 21) (see Figure 3, contrast 2 in the Table 2).

**Table 2:** Regions of significant activations.

Brain regions	BA	MNIcoordinates			t Value	Clustersize
		x	y	z		
**Main effect (SR>OR)**						
L. Medial frontal gyrus	10	−6	56	7	6.91	164
L. Anterior Cingulate	10	−12	50	1	6.58	
L. Anterior Cingulate	25	−3	35	4	5.99	
**Main effect (F>R)**						
L. Middle frontalgyrus(Rostral)	48	−48	21	23	7.63	657
L. Inferior frontal gyrus	47	−39	35	−2	7.59	
L. Superior parietal lobule	7	−30	−67	43	7	176
L. Inferior parietal lobule	40	−48	−43	52	6.06	
L. Inferior parietal lobule	40	−42	−49	52	5.92	
R. Cerebellum		27	−70	−29	7.41	162
R. Cerebellum		12	−79	−26	6.54	
L. Middle temporal gyrus	20	−57	−40	−8	6.22	57
L. Middle temporal gyrus	20	−54	−49	−11	6.05	
L. Middle temporal gyrus	21	−57	−49	1	5.3	
**Interaction**						
R. Cerebellum		27	−70	−29	5.59	46
R. Orbital frontal gyrus	47	−42	30	−4	5.47	15
L. Inferior frontal gyrus	44	−48	8	20	5.37	56
L. Middle frontalgyrus(Caudal)	44	−48	15	30	5.25	
**Simple effect (SR_F>R)**						
R. Cerebellum		25	−74	−30	5.21	73
R. Cerebellum		14	−79	−24	4.72	
L. Orbital frontal gyrus	47	−42	35	−2	5.47	41
L. Inferior frontal gyrus	45	−51	35	10	4.94	
L. Inferior frontal gyus	44	−48	11	25	5.41	135
L. Middle frontalgyrus(Caudal)	44	−48	11	34	5.3	
L. Middle frontalgyrus(Rostral)	48	−48	23	25	4.95	
**Simple effect (OR_F>R)**						
L. Middle frontalgyrus(Rostral)	48	−48	20	20	5.32	19

**Note:** F: forget instruction; R: remember instruction; SR: self reference; OR: Other reference; SR_F>R: the directed forgetting effect for the self-reference information; OR_F>R: the directed forgetting effect for the other-reference information; MNI: Montreal Neurological Institute. The significance level of the image threshold for fMRI data was first set to p<0.001(FDR corrected) for multiple comparisons with a threshold for minimum spatial extent of 10 contiguous voxels.

**Figure 4:**
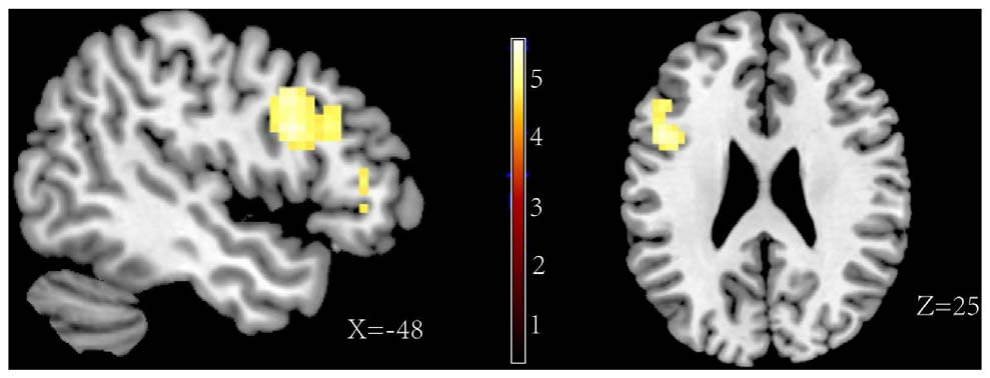
Activations of the forget instruction>remember instruction for the self-referential information.

**Figure 5:**
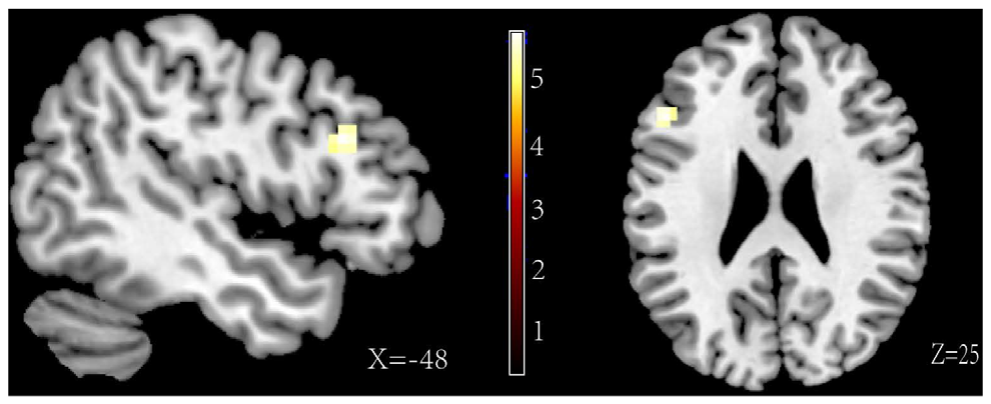
Activations of the forget instruction>remember instruction for the other-referential information.

**Figure 6:**
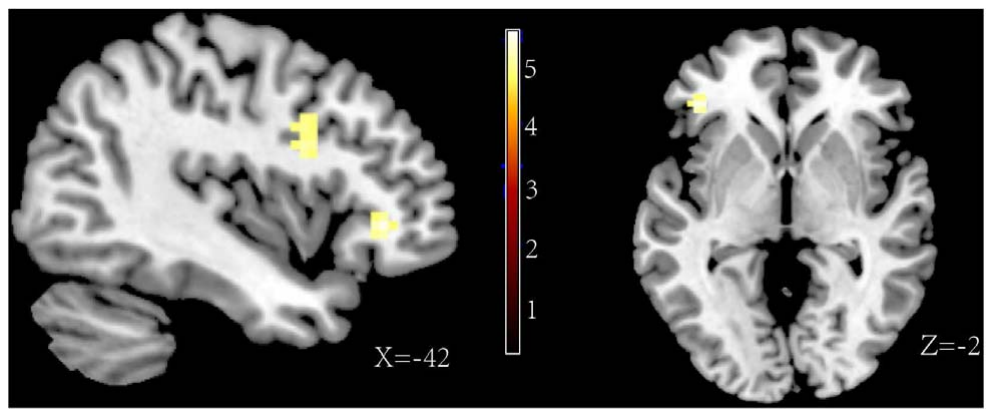
Activations of the interaction between the memory instruction and the reference type.

The major goal of this experiment was to explore the different neural correlations between forgetting self-referential negative memories and forgetting other-referential negative memories. Thus, the analyses directly contrasted the influence of the “F” and “R” instructions in separate trails of self-referential and other-referential information. For the self-referential information, the results of the F>R contrast revealed an activation of the orbital frontal gyrus (BA 47), the inferior frontal gyrus (BA 45, BA 44), and the middle frontal gyrus [including the caudal middle frontal gyrus (BA 44) and the rostral middle frontal gyrus (BA 48)] (see Figure 4, contrast 4 in Table 2). However, for the other-referential information, the result of the F>R contrast only showed an increased activity in the rostral middle frontal gyrus (BA 48) (see Figure 5, contrast 5 in Table 2). Consistent with these findings, the interaction reference×memory instruction showed a significant activation in the orbital frontal gyrus (BA 47), the inferior frontal gyrus (BA 44) and the caudal middle frontal gyrus (BA 44) (see Figure 6, contrast 3 in Table 2). The results confirmed that the specific activation of these areas for the directed forgetting effect in the self-referential information.

## Discussion

In this study, the directed forgetting paradigm was combined with the self-referential task to explore the difference between directed forgetting of self-referential memory and other-referential memory. Event-related fMRI data were obtained in the study phase when the memory instructions (F vs. R) were presented. The behavioral results were consistent with previous studies, which showed that a significant directed forgetting effect for both the self-referential and other-referential memory [27]. However, the highlight of this study was that a smaller directed forgetting effect was found for self-referential memory. The neural data showed that forgetting of the self-referential memories was correlated with a more widespread neural activation relative to forgetting of the other-referential memories. The results favored our hypothesis that intentional forgetting self-referential memory was more difficult than forgetting other-referential memory.

This study principally aimed to exam the differences between forgetting of the self-referential and other-referential memory. So, firstly we must ensure that the reference manipulation (self, other) is effective. The behavioral results showed that self-referential processing existed. At the neural level, the fMRI data showed that self-referential judgments resulted in stronger activity in the MPFC and ACC. These results were in line with the previous fMRI studies of SRE [31], [33], [35], [44], [45]. For example, Zhu et al. (2007) used the SRE task to explore the neural basis of culture-self in the Chinese and the Western participants. The results found that the MPFC and ACC showed stronger activation in self than other-judgment conditions for both Chinese and Western subjects. The MPFC maintained the abstract representation of the self, and the ACC reflect the participants think of their appearance and generate relevant emotional response. In our task, the stronger activity in the MPFC and ACC of the self-referential judgments relative to the other-referential judgments reflected that the participants made some self-reflective processes and relevant emotional responses. The present results indicated that our self reference manipulation was effective and participants made some special processing of the self-referential information.

The main effect of the memory instructions showed that, relative to the “R” instruction, the “F” instruction was associated with stronger activation in the middle frontal gyrus (BA 48), inferior frontal gyrus (BA 47), superior parietal lobule (BA 7), inferior parietal lobule (BA 40) and middle temporal gyrus (BA 20, BA 21) in the left hemisphere. A dominant feature of the result was that the frontal cortex was strongly activated by the “F” instruction. This feature was consistent with previous fMRI studies of directed forgetting [1], [17], [18]. Specifically, Wylie et al. (2007) used fMRI to explore the neural basis of the intentional remembering and forgetting. The results showed that compared with the intentional remembering and unintentional forgetting, the activation of the medial frontal gyrus was associated with the intentional forgetting. Nowicka et al. (2011) also found that the frontal activation was associated with the F instructions after the negative images. Meanwhile, some experiments used another memory control paradigm (TNT paradigm) also found that an attempt to suppress unwanted memory results in increased activation in the frontal gyrus [7], [8]. Based on the previous empirical data, we conclude that the frontal inhibitory control process is important in directed forgetting.

In addition to the frontal gyrus activation associated with the “F” instruction, the fMRI data also showed that the superior parietal lobule (BA 7), inferior parietal lobule (BA 40) and middle temporal gyrus (BA 20, BA 21) were activated when the “F” instruction was compared with the “R” instructions. Previous studies showed that the left inferior parietal areas and superior parietal region are parts of the attentional control network [46], [47]. The attentional networks engaged in our experiments suggested that directed forgetting depends on the involvement of attentional resources. The predominant left-lateralized activity was due to the verbal nature of the present task. Previous studies suggested that the lateralization of attention functions depend on whether the task is more verbal or spatially based. The verbal based tasks have been associated with left hemispheric activations, whereas visuospatial based tasks have typically been related to the right hemisphere [48], [49]. Previous results also showed that the medial temporal lobe has an important role in the active maintenance of novel information over working memory in the absence of perceptual stimulation [50]. When the “F” instructions were presented, the TBF items were not presented on the screen. The participants should first store the TBF items in the working memory and then initiate the frontal control mechanism to inhibit the rehearsal of TBF items or suppress their memory activation. Some experts also state that inhibition is postulated to be a mechanism by which the PFC exerts its effects on the sub-cortical and posterior-cortical regions to implement executive control [51]. In our study, the inhibitory control role of the prefrontal gyrus may exert some effects on the parietal gyrus and the temporal gyrus which associated with the attention and the working memory to inhibit the rehearsal of the TBF items.

The interesting result was that forgetting of the self-referential information is more difficult than forgetting of the other-referential information. The behavioral data showed that less self-referential memories were forgotten relative to other-referential memories. At the neural level, significant differences were found between directed forgetting of self-referential memories and other-referential memories. Higher activities in the orbital frontal gyrus (BA 47), the inferior frontal gyrus (BA 45, BA 44), and the middle frontal gyrus (BA 44, BA 48) were found in directed forgetting of self-referential memory. However, when forgetting of the other referential memories, only the rostral middle frontal gyrus (BA 48) was activated. Previous studies showed that the more difficult the task, the stronger the activations [41]. Specifically, some studies examined the effects of task difficulty in working memory and found that prefrontal and inferior frontal gyrus activation have been associated with increasing task difficulty [52], [53]. Gould et al. (2003) found that the left inferior frontal gyrus activated in the visuospatial learning task with increasing difficulty. Duncan and Owen (2000) reviewed activations in a range of tasks that had varied cognitive demand. They concluded that the medial gyrus and orbital frontal gyrus were activated under conditions of increasing cognitive demand. The recruitment of this particular activation network appears to be both task independent and stimuli independent [54]. In our study, higher inferior activation was associated with forgetting of the other-referential information relative to forgetting of the self-referential information. Combined the behavioral and fMRI data, we conclude that forgetting self-referential memories is a difficult task relative to forgetting other-referential memories.

The reason behind the different directed forgetting effects for self referential and other referential memories was also investigated. The attentional inhibition hypothesis of directed forgetting holds that directed forgetting results from the attentional inhibition of information during encoding. Specifically, the “F” instruction triggers the attentional inhibition that terminates the rehearsal of TBF items or suppresses their memory activation to below baseline levels (i.e., representational inhibition) [5], [13]. Based on the attentional inhibition hypothesis, less self-referential memories were forgotten because of attentional bias to self-referential information. It has been well documented that the self-related information seems to automatically attract the person’s attention [55]–[57]. In present study,self-referential processing information can capture the attention automatically, the attentional inhibition mechanism elicited by the “F” instruction is less efficient for the self-referential information than the other-referential ones.

In conclusion, this study compared the behavioral and neural mechanisms of directed forgetting of self-referential and other -referential memory. The behavioral data showed that little self-referential information can be forgotten than other-referential information. At the neural level, forgetting of the self-referential information was associated with more widespread activation relative to the other-referential information. The results indicated that though people can still forget negative self-referential information, it is more difficult than forgetting of the other-referential information. Participants had to make more effort to inhibit the negative self-referential memory with the inhibitory control mechanism. Future studies should focus on examining the effect of valence on directed forgetting of self-referential memory.
